# Breastfeeding and Postpartum Depression: An Overview and Methodological Recommendations for Future Research

**DOI:** 10.1155/2016/4765310

**Published:** 2016-04-11

**Authors:** Carley J. Pope, Dwight Mazmanian

**Affiliations:** Department of Psychology, Lakehead University, 955 Oliver Road, Thunder Bay, ON, Canada P7B 5E1

## Abstract

Emerging research suggests that a relationship exists between breastfeeding and postpartum depression; however, the direction and precise nature of this relationship are not yet clear. The purpose of this paper is to provide an overview of the relationship between breastfeeding and postpartum depression as it has been examined in the empirical literature. Also, the potential mechanisms of action that have been implicated in this relationship are also explored. PubMed and PsycINFO were searched using the keywords:* breastfeeding* with* postpartum depression, perinatal depression, postnatal depression*. Results of this search showed that researchers have examined this relationship in diverse ways using diverse methodology. In particular, researchers have examined the relationships between postpartum depression and breastfeeding intention, initiation, duration, and dose. Due to a number of methodological differences among past studies we make some recommendations for future research that will better facilitate an integration of findings. Future research should (1) use standardized assessment protocols; (2) confirm diagnosis through established clinical interview when possible; (3) provide a clear operationalized definition for breastfeeding variables; (4) clearly define the postpartum period interval assessed and time frame for onset of symptoms; (5) be prospective or longitudinal in nature; and (6) take into consideration other potential risk factors identified in the empirical literature.

## 1. Introduction

Postpartum depression is a serious mental health condition that affects an estimated 13% to 19% of women who have recently given birth [1]. Postpartum depression is characterized as a persistent low mood in new mothers, which is often accompanied by feelings of sadness, worthlessness, and/or hopelessness. Postpartum depression differs from the “baby blues,” as the “baby blues” is a briefer period of emotional disturbance (including dysphoria, tearfulness, mood lability, trouble sleeping, irritability, and anxiety) that is experienced by up to 4 in 5 women within the first few days following childbirth and usually remits within 10 days [1, 2].

Currently, the Diagnostic and Statistical Manual for Mental Disorders-Fifth Edition (DSM-5) classifies depression with peripartum onset as beginning during pregnancy or within the first four weeks postpartum [3]. The International Classification of Diseases (ICD) classifies postpartum depression as occurring within the first six weeks postpartum [4]. In contrast to the current criteria, some researchers recommend that this time frame be extended in future revisions of these guides to account for episode onset within the first six months postpartum [5]. Further, in spite of the current ICD and DSM-5 guidelines, many researchers use a time frame that ranges up to one year postpartum for onset of postpartum depression [1].

While the clinical profile of postpartum depression is similar to depression occurring at other times in a woman's life, it may differ in some respects due to the profound physiological changes occurring during pregnancy and the postpartum period [1, 6]. In fact, it is estimated that as many as 80% of postpartum women experience symptoms of mood disturbance within the first few days following childbirth [7]. Moreover, many postpartum women experience symptoms following pregnancy that are characteristic of depression, such as disturbances in appetite, energy, and sleep [8]. These factors make it difficult to differentiate onset of a clinically significant depressive disorder from common symptoms experienced as a result of childbirth and caring for a new infant.

While postpartum depression can be brief and remit unexpectedly, approximately 30% of women in community samples who experience postpartum depression continue to be depressed up to two years postpartum [9], and 50% of women from clinical samples continue to have major depression throughout, and in some cases beyond, the first year postpartum [10]. Furthermore, the illness course can vary and chronic depression for these women may consist of stable mild depression, stable major depression, or recurrent episodes of major depression without full remission between episodes [10].

## 2. Consequences of Postpartum Depression

Compared to depression occurring at other time points in a woman's life, there is some evidence to suggest that women experiencing postpartum depression are at an increased risk for comorbid obsessive compulsive disorder [11, 12] and anxiety [11, 13, 14]. For example, one study reported that 19.9% of women screening positive for postpartum depression at 2 weeks postpartum also screened positive for anxiety (compared to 1.3% of women who did not screen positive for postpartum depression; *p* < 0.001). In this same study, 25.7% of women screening positive for postpartum depression at 2 weeks postpartum also screened positive for obsessive compulsive symptoms (compared to 8.4% of women who did not screen positive for postpartum depression; *p* < 0.001). Suicidal ideation and thoughts of self-harm or thoughts of harming the infant are also reported to be more common in women with postpartum depression [15–18]. For instance, one study found that 30% of women who screened positive for postpartum depression endorsed at least some experience of thoughts of self-harm [17]. In another study, 41% of depressed mothers reported at least some thoughts regarding harming their infant compared to 7% of control mothers [15].

In addition to being at increased risk for comorbid disorders, postpartum depression is associated with numerous consequences in other domains [19]. Negative long-term consequences to the infant's social, emotional, cognitive, and physical development have been reported [20]. Children of mothers with a history of postpartum depression may also be at increased risk of developing psychosocial and emotional or behavioural disturbances [21], as well as intellectual disabilities [22]. Additionally, postpartum depression is associated with disturbance in mother-infant interactions and bonding, as well as deficient parenting and parental safety practices [20, 23]. Spinelli [24] has reported that infanticide is rare in women who are experiencing nonpsychotic postpartum depression, but infanticide is a possible serious and tragic consequence when postpartum depression is accompanied by psychosis. Although postpartum psychosis only occurs following one to two out of 1000 births [3], it can result in other serious and devastating consequences for the mother and the infant, as well as the rest of the family [25]. Often postpartum psychosis leads to hospitalization and considerable functional impairment [26]. There is, however, some debate concerning the degree to which psychosis is related to the unipolar variation of postpartum depression [24]. Emerging evidence suggests that postpartum psychosis is more likely a variant of bipolar disorder [27].

Due to the potential devastating consequences of postpartum depression for the mother, the infant, and their family, it is necessary that research clearly delineate the potential risk and protective factors for postpartum depression. Emerging research suggests that breastfeeding may offer protective benefits against postpartum depression [28]; however, the exact nature of the association between breastfeeding and postpartum depression remains unclear [29]. The purpose of this paper is to provide an overview of the association between breastfeeding and postpartum depression as it has been examined in the empirical literature. To conduct this review we searched PubMed and PsycINFO using the keywords:* breastfeeding* with* postpartum depression, perinatal depression, postnatal depression*. All original studies located through this search and reported in English examining the relationship between postpartum depression and breastfeeding were included.

## 3. Breastfeeding and Postpartum Depression

Initially the relationship between breastfeeding and postpartum depression was conceptualized to be unidirectional, with postpartum depression resulting in lower rates of breastfeeding initiation and early cessation [30]. More recently however, reports indicate that the relationship may be bidirectional in nature, suggesting that while postpartum depression may reduce rates of breastfeeding, not engaging in breastfeeding may increase the risk of postpartum depression. Additionally, there is some evidence that breastfeeding may protect against postpartum depression or assist in a swifter recovery from symptoms [28].

The association between breastfeeding and postpartum depression has been studied by a number of investigators, but the direction of this relationship and the question of whether it is a direct relationship still eludes us. Numerous studies on the topic of breastfeeding and postpartum depression have come to contrasting conclusions, likely a result of the interaction between the numerous and complex physiological, psychological, and sociocultural mechanisms potentially responsible for the relationship [31], as well as the use of varying methods for studying the association. Specifically, a number of researchers have reported no relationship between breastfeeding and postpartum depression (e.g., see [32]) and two early reports suggested that breastfeeding mothers have a higher risk of depression [33, 34]. In contrast, a number of more recent studies have revealed that women who formula feed have higher rates of depression than women who breastfeed (e.g., see [35]), while other researchers have shown that mothers who experience postpartum depression are at greater risk of early breastfeeding cessation (e.g., see [36]).

## 4. Breastfeeding Intention and Initiation

Intention to breastfeed refers to the perinatal decision to breastfeed an infant following childbirth, but the prenatal time frame may differ between studies depending on when this information was obtained (i.e., prospectively or retrospectively). Breastfeeding initiation refers broadly to the act of breastfeeding. Unfortunately, breastfeeding initiation is operationalized differently between investigations and may include women who attempt to breastfeed but discontinue shortly thereafter, women who exclusively breastfeed for long periods, women who used expressed breast milk, and women who supplement their infant with formula in addition to breastfeeding. These discrepancies can complicate our interpretation of the research. With that being said, a number of investigators have examined the relationship between breastfeeding intention or initiation and postpartum depression.

Some researchers have found no association between prenatal depressive symptoms and intention to breastfeed [37–40]. In contrast, Insaf and colleagues [41] did find that women with prenatal depressive symptoms were less likely to intend to breastfeed, though this study did not follow the women through to childbirth to determine initiation rates. Similarly, Fairlie and colleagues [42] also found that prenatal depressive symptoms were associated with a reduced intention to breastfeed (reported in the second trimester). However, follow-up in the postpartum period revealed that depressive symptoms during pregnancy were not associated with the actual initiation of breastfeeding, indicating that some of the women who initially reported that they did not intend to breastfeed changed their minds and attempted to breastfeed. Furthermore, Pippins and colleagues [43] found, in their longitudinal study following a large sample of pregnant women, that women with prenatal depressive symptoms were not less likely to initiate breastfeeding. Thus, it appears that women's prenatal intention to breastfeed may fluctuate, perhaps due to breastfeeding encouragement or education in the third trimester.

The potential relationship between breastfeeding intention and initiation and postpartum depression was further explored by Borra et al. [44]. In this large longitudinal study the investigators found that, in women who were not depressed before delivery, the risk of postpartum depression was decreased if they had intended to breastfeed and initiated breastfeeding. In contrast, women were at an increased risk for postpartum depression if they had not intended to breastfeed and initiated breastfeeding. Interestingly, Davey and colleagues [45] found that failure to breastfeed (when attempted) is associated with postpartum depressive symptoms. Also, women who never established breastfeeding were reported to have a 2.4-fold chance of developing depressive symptoms at 16 weeks postpartum compared to breastfeeding women [46].

## 5. Breastfeeding and Maternal Mood

A number of studies report that women who are not breastfeeding are more likely to have higher levels of depressive symptoms than women who are breastfeeding [35, 47–61]. For example, a recent longitudinal study by Nishioka and colleagues [57] found that at 5 months postpartum the proportion of mothers with an Edinburgh Postnatal Depression Scale [62] score of ≥9 (suggesting risk of postpartum depression) was significantly lower for women who were breastfeeding compared to women who were formula feeding (*p* = 0.04). Moreover, the inverse relationship between breastfeeding and postpartum depressive symptoms was found to persist even once age and education were controlled (OR = 0.28, *p* = 0.007) [63] and when income, race, previous history of depression, or current psychoactive medication use was controlled (*p* < 0.001) [64]. Interestingly, one study found that depression severity was not related to breastfeeding status in a group of women diagnosed with postpartum depression [65]. Thus, while breastfeeding may be associated with depressive symptoms, it may not influence the severity of the symptoms.

Although postpartum depression has been identified as a risk factor for early breastfeeding cessation [56], early negative breastfeeding experiences may be a risk factor for postpartum depression [66]. Further, it has also been suggested that breastfeeding may offer protective benefits against postpartum depression [28]. One study to report on the protective benefits of breastfeeding found that lower levels of depressive symptoms in the prenatal but not postnatal period predicted exclusive breastfeeding. Furthermore, breastfeeding duration was associated with a significant decrease in depressive symptom scores from childbirth to 3 months postpartum for women who initiated breastfeeding. These investigators also found that women who did not initiate breastfeeding did not experience changes in depressive symptoms over the first three postpartum months. After considering the findings collectively, the investigators postulated that breastfeeding alleviates depressive symptomology over time [28].

Additionally, results from a study by Mezzacappa and Katkin [67] lend further support to the premise that breastfeeding offers ameliorating effects on postpartum depressive mood symptoms. These investigators looked at the acute effects of breastfeeding on maternal mood and found that breastfeeding mothers experienced a decrease in negative mood from prefeeding to postfeeding. Moreover, bottle-feeding mothers experienced a decrease in positive mood from prefeeding to postfeeding. Thus, breastfeeding may offer both acute and long-term ameliorating effects on postpartum depression; however, further research is required to substantiate these initial findings.

## 6. Breastfeeding Duration

Breastfeeding duration has been found to be inversely related to postpartum depressive symptoms. A relationship has been found to persist even after controlling for socioeconomic status, age, and education level [68], as well as for past history of depression, increased life stress, and psychoactive medication use [64]. In particular, a number of studies have reported an association between postpartum depressive symptoms and early weaning [30, 69–75]. In fact, McLearn et al. [56] reported that mothers with depressive symptoms were less likely to continue breastfeeding through to two to four months postpartum compared to mothers without depressive symptoms (AOR = 0.73, *p* < 0.001).

A number of studies note that postpartum depressive symptoms preceded breastfeeding cessation [68, 76]. In a large prospective study of postpartum women, Taveras and colleagues [77] found that having higher depressive symptoms at two weeks postpartum was associated with discontinuation of breastfeeding at 12 weeks postpartum. Dennis and McQueen [36] reported similar findings. Specifically, depressive symptomology in the early postpartum period predicted early cessation of breastfeeding at eight weeks postpartum. Also, in a smaller prospective study, Galler et al. [78] found that depressive symptoms at seven weeks postpartum inversely predicted breastfeeding practices at seven weeks, three months, and six months postpartum, even after controlling for disadvantaged environmental conditions. Interestingly, these investigators did not find an association between depressive symptoms at six months postpartum and breastfeeding practices at the same time point.

More recently, Dennis and McQueen [36] found that after controlling for baseline depressive symptoms there was no relationship between infant feeding outcome (feeding method used, satisfaction with method, breastfeeding difficulties, and breastfeeding self-efficacy) at one week postpartum and the development of postpartum depressive symptoms (measured one and two months postpartum). However, the women in this study who reported high levels of postpartum depressive symptoms were significantly more likely to discontinue breastfeeding. These women were also more likely to report being unsatisfied with their infant-feeding method, experience breastfeeding difficulties, and report lower breastfeeding self-efficacy. Taken together, these findings suggested that over time depressive symptoms may influence breastfeeding outcomes to a point of discontinuation.

## 7. Breastfeeding Dose-Response Effect

A dose-response effect of breastfeeding on postpartum depression has been proposed. In a large study of women evaluated between 8 and 12 weeks postpartum, Thome et al. [79] found that exclusively breastfeeding mothers had lower mean depressive symptom scores compared to partial breastfeeding mothers. Relatedly, Ystrom [80] found that, at six months postpartum, both partially breastfeeding and exclusively bottle-feeding were significantly related to higher levels of depressive symptoms in postpartum women compared to those who exclusively breastfed. Furthermore, bottle-feeding was related to postpartum depression to a greater degree than partial breastfeeding. Also, when the investigator adjusted for baseline prenatal anxiety and depression (measured at 30 weeks of gestation) the relationship persisted, indicating that breastfeeding may reduce depressive symptoms or depressive symptoms may result in breastfeeding titration.

One other study compared exclusive breastfeeding to exclusive bottle-feeding [81]. These investigators found an inverse association between postpartum depression and exclusive breastfeeding continuation. Also, it has been found that as early as one week postpartum levels of depressive symptoms are inversely related to exclusive breastfeeding [82]. Moreover, Kendall-Tackett et al. [83] investigated the effects of breastfeeding on women at high risk for postpartum depression and sleep difficulties due to a history of being victims of sexual assault. These investigators reported a relationship between exclusive breastfeeding and fewer reported sleep difficulties and depressive symptoms compared to women who partially breastfed or bottle-fed their infant. Unfortunately these results are based on analyses of cross-sectional data, making the direction of the relationship in each case unclear.

## 8. Reciprocal Relational Findings

In light of conflicting reports that postpartum depression leads to early breastfeeding cessation (e.g., see [56]) and that breastfeeding leads to a reduction in postpartum depressive symptoms (e.g., see [67]), recent investigations have examined the possible reciprocal relationship. Specifically, it has been proposed that postpartum depression can lead to early breastfeeding cessation but breastfeeding continuation may also reduce levels of postpartum depressive symptoms [28]. For example, results of a study by Hamdan and Tamim [31] support the reciprocal relationship hypothesis. These investigators found that women who were breastfeeding at two months postpartum had a lower risk of postpartum depression at four months postpartum. On the other hand, women who had postpartum depression at two months postpartum were less likely to be breastfeeding at four months postpartum. Also, Hahn-Holbrook et al. [84] found that prenatal depressive symptoms predicted a reduced frequency of breastfeeding and earlier cessation within the first three months postpartum. These researchers also found that more frequent breastfeeding at three months postpartum was associated with greater subsequent declines in depressive symptom levels up to two years postpartum.

## 9. No Association or Nonsignificant Trends

A number of studies have reported no significant relationship between postpartum depression and breastfeeding status [32, 39, 85–91]. However, two of these studies did report finding a nonsignificant trend suggestive of an inverse association [86, 89]. In any case, most of these findings were incidental; the primary purpose of those investigations was not to evaluate the association between breastfeeding and postpartum depression. However, recent data analyzed in our lab also failed to find support for a relationship between breastfeeding intention and initiation when controlling for other risk factors for postpartum depression.

## 10. Conflicting Research Findings

A majority of studies do report some association between breastfeeding and postpartum depression; however the direction of the relationship is unclear and some of the findings conflict with one another. This is likely a reflection of both the complex processes responsible for the association between breastfeeding and postpartum depression and the differences between the study designs and the samples used. Dennis and McQueen [92] suggested that the contrasting findings may be due to differences in research methodology or study limitations. Some of the limitations include differences in definition and criteria for assessing breastfeeding. That is, a number of studies only classified breastfeeding as a “yes” or “no” dichotomy, failing to take into account partial breastfeeding (e.g., see [32, 39, 87, 88, 90]). Also, one of the methodological differences that makes integration and comparison of studies difficult pertains to various discrepancies in how postpartum depression is operationalized between studies. For instance, some studies used assessment instruments that were not specific to depression (e.g., see [87]) or used lower cut-off scores (e.g., see [88, 89]) compared to most investigations. Furthermore, some studies used samples with higher than normal rates of women reporting postpartum depression. For instance, Lau and Chan [89] found the rate of postpartum depression in their sample to be 34%, which is about double the estimated prevalence [1], likely a result of the low cut-off score they used. Also, some studies used women at high risk for postpartum depression. For instance, Kendall-Tackett et al.'s [83] investigation used women who reported a history of sexual assault, a proposed risk factor for postpartum depression [10]. Furthermore, for many of the studies, the primary focus was not to delineate the relationship between breastfeeding and depression [92], which likely explains many of the methodological or interpretive shortfalls noted.

## 11. Mechanism of Action

The mechanisms by which breastfeeding is affected by or affects postpartum depression have been assessed in a number of studies. Breastfeeding self-efficacy and negative breastfeeding perceptions have been implicated as playing a primary role in the relationship. Specifically, during the first week postpartum, depressed mothers have been found to be at increased risk for feeling unsatisfied with breastfeeding and were experiencing significant breastfeeding problems. They are also at risk for experiencing lower levels of breastfeeding self-efficacy compared to nondepressed mothers [36].

Furthermore, mothers' postpartum depressive symptoms were found in one study to be inversely related to the belief that breastfeeding is the best option for infant feeding and positively related to the beliefs that breastfeeding is private and breastfeeding is restrictive [93]. Additionally, in a study that did not find a relationship between breastfeeding and depressive symptoms, women who worried about breastfeeding were significantly more likely to develop depression than women who did not worry [32]. Similarly, Tamminen [94] found that women with more depressive symptoms also reported more breastfeeding difficulties, and Dennis [95] noted that level of depressive symptoms at one, four, and eight weeks postpartum was inversely related to breastfeeding self-efficacy at the corresponding time periods. This is a similar finding to that recorded by Dai and Dennis [96]. Collectively, this research suggests that it is not necessarily the postpartum depression per se that leads to reduced breastfeeding; it might be the mothers' negative perceptions of their breastfeeding experiences that are responsible.

Alternatively, complications with the mother-infant interaction may also play a role. One study reported that breastfeeding led to less burping, less intrusive stimulation (e.g., mother poking the infant or moving) during nipple-in and nipple-out periods, and more stroking (by the mother to the infant) as well as superior mother-infant interaction rating scores as rated by an observer. Further, these benefits were found to extend to both the depressed and nondepressed breastfeeding women [97]. Thus, breastfeeding may enhance the mother-child interaction, which may lead to improved maternal mental health.

Breastfeeding difficulties and lack of breastfeeding confidence are reported as common concerns for mothers with postpartum depressive symptoms [98]. Dennis and McQueen [36] suggested that the factors underlying the relationship between breastfeeding duration and postpartum depression are multifactorial. In other words, it is likely that the interplay between the mothers' negative cognitions and impaired mother-infant interaction, in addition to other factors, such as underlying physiological processes, are responsible for the emergence of postpartum depression. Moreover, depressive symptoms in the early postpartum period resulted in the mother being more vulnerable to feelings of low self-esteem and self-efficacy. As a consequence, the depressive symptoms and accompanying negative cognitions may reinforce perceived breastfeeding difficulties or may reduce the mothers' ability to accurately interpret infant cues, further perpetuating actual breastfeeding difficulties [36].

Also, breastfeeding is suggested to attenuate neuroendocrine responses to stress and may act to enhance maternal mood. Specifically, oxytocin and prolactin, hormones responsible for lactation, are suggested to have mood-ameliorating effects. Oxytocin in particular is a hormone that promotes feelings of nurturance and relaxation during nursing [99–101]. Also, lactation is suggested to attenuate cortisol stress responses [102] by decreasing stress hormone levels (especially cortisol) and enhancing sleep [103]. In addition to enhancing mood, a number of neuroendocrine mechanisms have been implicated in both breastfeeding failure and postpartum depression including gonadal/placental steroids and lactogenic hormones, as well as neuroendocrine pathways related to stress reactivity [104]. Moreover, it is also possible that only a subset of women who have a hormonal sensitivity are at risk for depressive symptoms in relation to breastfeeding cessation. Thus, future research aimed at assessing the specific circumstances whereby breastfeeding may offer protective or ameliorating benefits against postpartum depression is warranted and needed in order to make informed recommendations tailored to each new mothers' unique circumstance.

## 12. Future Research

Future researchers examining the relationship between breastfeeding and postpartum depression should consider the following methodological recommendations. These recommendations are made in an effort to make results of this research more comparable and to allow for an integration of findings to aid in conclusions and clinical recommendations. Future researchers should use standardized assessment protocols and cut-off values established by psychometric evaluations of the measures. Currently the Edinburgh Postnatal Depression Scale (EPDS) is among the most widely used empirically validated self-report screening measures for postpartum depression. Using a cut-off value of 13 or greater, this measure is reported to have a sensitivity of 68–86%, specificity of 78–96%, and positive predictive value of 67–73% [62, 105]. When possible, diagnostic clarification through the use of established structured clinical interviews is ideal as this process is currently the gold standard for psychiatric diagnosis. Use of both the EPDS and diagnostic interviews can be valuable as each provides a different approach to examining the relationship between breastfeeding and postpartum depression. That is, information from the EPDS allows researchers to evaluate depressive symptoms on a continuum to examine whether breastfeeding status might be associated with more transient symptoms or subclinical levels of depression. The addition of a diagnostic interview allows researchers to evaluate whether breastfeeding is involved in clinically confirmed postpartum depression.

Future researchers should also specify and clearly define breastfeeding variables as well as the time intervals assessed for postpartum depression. Investigators should also make efforts to determine the time frame for onset of symptoms. Currently there is debate over the time frame at which a woman is considered to have onset of a postpartum depressive episode [1]. Diagnostic guidelines dictate time frames ranging from anytime during pregnancy to the first six weeks postpartum [3, 4]. However, many researchers and clinicians consider onset within the first year postpartum to be considered a postpartum depressive episode [1, 5]. Due to the lack of consensus regarding what constitutes postpartum onset and variation in the postpartum intervals investigated by researchers it is important that investigators are very clear about the interval addressed in their research. This is especially pertinent as various biological, environmental, and psychosocial factors that may influence the relationship between breastfeeding and postpartum depression can vary depending on the postpartum time period being assessed.

Research that is prospective or longitudinal in nature will also be helpful in evaluating the direction of the proposed relationship between breastfeeding and postpartum depression. This will facilitate inferences regarding the temporal relationship. Such designs should take into consideration other potential factors suggested to influence postpartum depression. Considering the role of other potential postpartum depression risk factors within the breastfeeding and postpartum depression dynamic will better facilitate interpretation of results. Some of the risk factors more commonly identified in research include low socioeconomic status [1, 31], poor social support [46], and history of depression [31, 43, 45, 53].

## 13. Conclusions

The primary purpose of this paper was to provide an overview of the potential relationships between breastfeeding and postpartum depression that have been suggested by research and to provide recommendations to facilitate comparisons between investigations. Due to numerous methodological discrepancies between studies it is difficult to draw conclusions at this time. Also, interpretations of research are impeded by many of the same conundrums that exist when attempting to empirically understand postpartum depression in general. For instance, much of the research is naturalistic in nature, restricting the ability to make causal inferences. Also, the physiological changes that occur over the course of pregnancy and the postpartum period are not completely understood and hormonal shifts during the postpartum period may influence women's mental health and well-being in different ways depending on the time period referenced. Improved standardization between future research investigations will promote comparison between studies. To facilitate comparison and integration of study findings in this area we recommend that future research protocols (1) use standardized assessment protocols; (2) confirm diagnosis through established clinical interview when possible; (3) provide a clear operationalized definition for breastfeeding variables; (4) clearly define the postpartum period interval assessed and time frame for onset of symptoms; (5) be prospective or longitudinal in nature; and (6) take into consideration other potential risk factors identified in the empirical literature.

## References

[1] O'Hara M. W., McCabe J. E. (2013). Postpartum depression: current status and future directions. *Annual Review of Clinical Psychology*.

[2] Heron J., Haque S., Oyebode F., Craddock N., Jones I. (2009). A longitudinal study of hypomania and depression symptoms in pregnancy and the postpartum period. *Bipolar Disorders*.

[3] American Psychiatric Association (2013). *Diagnostic and Statistical Manual of Mental Disorders*.

[4] World Health Organization (2010). *The ICD-10 Classification of Mental and Behavioural Disorders*.

[5] Sharma V., Mazmanian D. (2014). The DSM-5 peripartum specifier: prospects and pitfalls. *Archives of Women's Mental Health*.

[6] Bloch M., Schmidt P. J., Danaceau M., Murphy J., Nieman L., Rubinow D. R. (2000). Effects of gonadal steroids in women with a history of postpartum depression. *The American Journal of Psychiatry*.

[7] Buttner M. M., O'Hara M. W., Watson D. (2012). The structure of women's mood in the early postpartum. *Assessment*.

[8] O'Hara M. W., Schlechte J. A., Lewis D. A., Wright E. J. (1991). Prospective study of postpartum blues: biologic and psychosocial factors. *Archives of General Psychiatry*.

[9] Horowitz J. A., Goodman J. (2004). A longitudinal study of maternal postpartum depression symptoms. *Research and Theory for Nursing Practice*.

[10] Vliegen N., Casalin S., Luyten P. (2014). The course of postpartum depression: a review of longitudinal studies. *Harvard Review of Psychiatry*.

[11] Miller E. S., Hoxha D., Wisner K. L., Gossett D. R. (2014). The impact of perinatal depression on the evolution of anxiety and obsessive-compulsive symptoms. *Archives of Women's Mental Health*.

[12] Russell E. J., Fawcett J. M., Mazmanian D. (2013). Risk of obsessive-compulsive disorder in pregnant and postpartum women: a meta-analysis. *Journal of Clinical Psychiatry*.

[13] Hendrick V., Altshuler L., Strouse T., Grosser S. (2000). Postpartum and nonpostpartum depression: differences in presentation and response to pharmacologic treatment. *Depression and Anxiety*.

[14] O'Brien M., Buikstra E., Hegney D. (2008). The influence of psychological factors on breastfeeding duration. *Journal of Advanced Nursing*.

[15] Jennings K. D., Ross S., Popper S., Elmore M. (1999). Thoughts of harming infants in depressed and nondepressed mothers. *Journal of Affective Disorders*.

[16] Pope C. J., Xie B., Sharma V., Campbell M. K. (2013). A prospective study of thoughts of self-harm and suicidal ideation during the postpartum period in women with mood disorders. *Archives of Women's Mental Health*.

[17] Wisner K. L., Sit D. K. Y., McShea M. C. (2013). Onset timing, thoughts of self-harm, and diagnoses in postpartum women with screen-positive depression findings. *JAMA Psychiatry*.

[18] Lindahl V., Pearson J. L., Colpe L. (2005). Prevalence of suicidality during pregnancy and the postpartum. *Archives of Women's Mental Health*.

[19] Pope C. J., Sharma V., Mazmanian D. (2014). Recognition, diagnosis and treatment of postpartum bipolar depression. *Expert Review of Neurotherapeutics*.

[20] Field T. (2010). Postpartum depression effects on early interactions, parenting, and safety practices: a review. *Infant Behavior & Development*.

[21] Korhonen M., Luoma I., Salmelin R., Tamminen T. (2012). A longitudinal study of maternal prenatal, postnatal and concurrent depressive symptoms and adolescent well-being. *Journal of Affective Disorders*.

[22] Morgan V. A., Croft M. L., Valuri G. M. (2012). Intellectual disability and other neuropsychiatric outcomes in high-risk children of mothers with schizophrenia, bipolar disorder and unipolar major depression. *The British Journal of Psychiatry*.

[23] Moehler E., Brunner R., Wiebel A., Reck C., Resch F. (2006). Maternal depressive symptoms in the postnatal period are associated with long-term impairment of mother-child bonding. *Archives of Women's Mental Health*.

[24] Spinelli M. G. (2004). Maternal infanticide associated with mental illness: prevention and the promise of saved lives. *The American Journal of Psychiatry*.

[25] Ganjekar S., Desai G., Chandra P. S. (2013). A comparative study of psychopathology, symptom severity, and short-term outcome of postpartum and nonpostpartum mania. *Bipolar Disorders*.

[26] Robertson E., Jones I., Haque S., Holder R., Craddock N. (2005). Risk of puerperal and non-puerperal recurrence of illness following bipolar affective puerperal (post-partum) psychosis. *The British Journal of Psychiatry*.

[27] Brockington I. (2004). Postpartum psychiatric disorders. *The Lancet*.

[28] Figueiredo B., Canário C., Field T. (2014). Breastfeeding is negatively affected by prenatal depression and reduces postpartum depression. *Psychological Medicine*.

[29] Dias C. C., Figueiredo B. (2015). Breastfeeding and depression: a systematic review of the literature. *Journal of Affective Disorders*.

[30] Seimyr L., Edhborg M., Lundh W., Sjögren B. (2004). In the shadow of maternal depressed mood: experiences of parenthood during the first year after childbirth. *Journal of Psychosomatic Obstetrics and Gynecology*.

[31] Hamdan A., Tamim H. (2012). The relationship between postpartum depression and breastfeeding. *International Journal of Psychiatry in Medicine*.

[32] Chaudron L. H., Klein M. H., Remington P., Palta M., Allen C., Essex M. J. (2001). Predictors, prodromes and incidence of postpartum depression. *Journal of Psychosomatic Obstetrics & Gynecology*.

[33] Alder E. M., Cox J. L. (1983). Breast feeding and post-natal depression. *Journal of Psychosomatic Research*.

[34] Alder E., Bancroft J. (1988). The relationship between breast feeding persistence, sexuality and mood in postpartum women. *Psychological Medicine*.

[35] Groër M. W. (2005). Differences between exclusive breastfeeders, formula-feeders, and controls: a study of stress, mood, and endocrine variables. *Biological Research for Nursing*.

[36] Dennis C.-L., McQueen K. (2007). Does maternal postpartum depressive symptomatology influence infant feeding outcomes?. *Acta Paediatrica*.

[37] Barnes J., Stein A., Smith T., Pollock J. I. (1997). Extreme attitudes to body shape, social and psychological factors and a reluctance to breast feed. ALSPAC Study Team. Avon Longitudinal Study of Pregnancy and Childhood. *Journal of the Royal Society of Medicine*.

[38] Jacobson S. W., Jacobson J. L., Frye K. F. (1991). Incidence and correlates of breast-feeding in socioeconomically disadvantaged women. *Pediatrics*.

[39] McKee M. D., Zayas L. H., Jankowski K. R. B. (2004). Breastfeeding intention and practice in an urban minority population: relationship to maternal depressive symptoms and mother-infant closeness. *Journal of Reproductive and Infant Psychology*.

[40] Lee H. J., Rubio M. R., Elo I. T., McCollum K. F., Chung E. K., Culhane J. F. (2005). Factors associated with intention to breastfeed among low-income, inner-city pregnant women. *Maternal and Child Health Journal*.

[41] Insaf T. Z., Fortner R. T., Pekow P., Dole N., Markenson G., Chasan-Taber L. (2011). Prenatal stress, anxiety, and depressive symptoms as predictors of intention to breastfeed among Hispanic women. *Journal of Women's Health*.

[42] Fairlie T. G., Gillman M. W., Rich-Edwards J. (2009). High pregnancy-related anxiety and prenatal depressive symptoms as predictors of intention to breastfeed and breastfeeding initiation. *Journal of Women's Health*.

[43] Pippins J. R., Brawarsky P., Jackson R. A., Fuentes-Afflick E., Haas J. S. (2006). Association of breastfeeding with maternal depressive symptoms. *Journal of Women's Health*.

[44] Borra C., Iacovou M., Sevilla A. (2015). New evidence on breastfeeding and postpartum depression: the importance of understanding women’s intentions. *Maternal and Child Health Journal*.

[45] Davey H. L., Tough S. C., Adair C. E., Benzies K. M. (2011). Risk factors for sub-clinical and major postpartum depression among a community cohort of canadian women. *Maternal and Child Health Journal*.

[46] Nielsen Forman D., Videbech P., Hedegaard M., Salvig J. D., Secher N. J. (2000). Postpartum depression: identification of women at risk. *BJOG: An International Journal of Obstetrics & Gynaecology*.

[47] Abou-Saleh M., Ghubash R., Karim L., Krymski M., Bhai I. (1998). Hormonal aspects of postpartum depression. *Psychoneuroendocrinology*.

[48] Astbury J., Brown S., Lumley J., Small R. (1994). Birth events, birth experiences and social differences in postnatal depression. *Australian Journal of Public Health*.

[49] Green K., Broome H., Mirabella J. (2006). Postnatal depression among mothers in the United Arab Emirates: socio-cultural and physical factors. *Psychology, Health and Medicine*.

[50] Groer M. W., Morgan K. (2007). Immune, health and endocrine characteristics of depressed postpartum mothers. *Psychoneuroendocrinology*.

[51] Gross K. H., Wells C. S., Radigan-Garcia A., Dietz P. M. (2002). Correlates of self-reports of being very depressed in the months after delivery: results from the pregnancy risk assessment monitoring system. *Maternal and child health journal*.

[52] Hannah P., Adams D., Lee A., Glover V., Sandler M. (1992). Links between early post-partum mood and post-natal depression. *The British Journal of Psychiatry*.

[53] Jardri R., Pelta J., Maron M. (2006). Predictive validation study of the Edinburgh postnatal depression scale in the first week after delivery and risk analysis for postnatal depression. *Journal of Affective Disorders*.

[54] Lane A., Keville R., Morris M., Kinsella A., Turner M., Barry S. (1997). Postnatal depression and elation among mothers and their partners: prevalence and predictors. *The British Journal of Psychiatry*.

[55] Mancini F., Carlson C., Albers L. (2007). Use of the postpartum depression screening scale in a collaborative obstetric practice. *Journal of Midwifery and Women's Health*.

[56] McLearn K. T., Minkovitz C. S., Strobino D. M., Marks E., Hou W. (2006). Maternal depressive symptoms at 2 to 4 months post partum and early parenting practices. *Archives of Pediatrics and Adolescent Medicine*.

[57] Nishioka E., Haruna M., Ota E. (2011). A prospective study of the relationship between breastfeeding and postpartum depressive symptoms appearing at 1-5 months after delivery. *Journal of Affective Disorders*.

[58] Tammentie T., Tarkka M.-T., Åstedt-Kurk P., Paavilainen E. (2002). Sociodemographic factors of families related to postnatal depressive symptoms of mothers. *International Journal of Nursing Practice*.

[59] Tashakori A., Behbahani A. Z., Irani R. D. (2012). Comparison of prevalence of postpartum depression symptoms between breastfeeding mothers an non-breastfeeding mothers. *Iranian Journal of Psychiatry*.

[60] Warner R., Appleby L., Whitton A., Faragher B. (1996). Demographic and obstetric risk factors for postnatal psychiatric morbidity. *The British Journal of Psychiatry*.

[61] Yonkers K. A., Ramin S. M., Rush A. J. (2001). Onset and persistence of postpartum depression in an inner-city maternal health clinic system. *The American Journal of Psychiatry*.

[62] Cox J. L., Holden J. M., Sagovsky R. (1987). Detection of postnatal depression: development of the 10-item edinburgh postnatal depression scale. *British Journal of Psychiatry*.

[63] Dunn S., Davies B., McCleary L., Edwards N., Gaboury I. (2006). The relationship between vulnerability factors and breastfeeding outcome. *Journal of Obstetric, Gynecologic, & Neonatal Nursing*.

[64] Hatton D. C., Harrison-Hohner J., Coste S., Dorato V., Curet L. B., McCarron D. A. (2005). Symptoms of postpartum depression and breastfeeding. *Journal of Human Lactation*.

[65] McCarter-Spaulding D., Horowitz J. A. (2007). How does postpartum depression affect breastfeeding?. *MCN The American Journal of Maternal/Child Nursing*.

[66] Watkins S., Meltzer-Brody S., Zolnoun D., Stuebe A. (2011). Early breastfeeding experiences and postpartum depression. *Obstetrics & Gynecology*.

[67] Mezzacappa E. S., Katkin E. S. (2002). Breast-feeding is associated with reduced perceived stress and negative mood in mothers. *Health Psychology*.

[68] Henderson J. J., Evans S. F., Straton J. A. Y., Priest S. R., Hagan R. (2003). Impact of postnatal depression on breastfeeding duration. *Birth*.

[69] Akman I., Kuscu M. K., Yurdakul Z. (2008). Breastfeeding duration and postpartum psychological adjustment: role of maternal attachment styles. *Journal of Paediatrics and Child Health*.

[70] Bick D. E., MacArthur C., Lancashire R. J. (1998). What influences the uptake and early cessation of breast feeding?. *Midwifery*.

[71] Cooper P. J., Murray L., Stein A. (1993). Psychosocial factors associated with the early termination of breast-feeding. *Journal of Psychosomatic Research*.

[72] Falceto O. G., Giugliani E. R. J., Fernandes C. L. C. (2004). Influence of parental mental health on early termination of breast-feeding: a case-control study. *Journal of the American Board of Family Practice*.

[73] Fergerson S. S., Jamieson D. J., Lindsay M. (2002). Diagnosing postpartum depression: can we do better?. *American Journal of Obstetrics & Gynecology*.

[74] Papinczak T. A., Turner C. T. (2000). An analysis of personal and social factors influencing initiation and duration of breastfeeding in a large Queensland maternity hospital. *Breastfeeding Review*.

[75] Pearlstein T., Howard M., Salisbury A., Zlotnick C. (2009). Postpartum depression. *American Journal of Obstetrics & Gynecology*.

[76] Misri S., Sinclair D. A., Kuan A. J. (1997). Breast-feeding and postpartum depression: is there a relationship?. *The Canadian Journal of Psychiatry*.

[77] Taveras E. M., Capra A. M., Braveman P. A., Jensvold N. G., Escobar G. J., Lieu T. A. (2003). Clinician support and psychosocial risk factors associated with breastfeeding discontinuation. *Pediatrics*.

[78] Galler J. R., Harrison R. H., Biggs M. A., Ramsey F., Forde V. (1999). Maternal moods predict breastfeeding in Barbados. *Journal of Developmental and Behavioral Pediatrics*.

[79] Thome M., Alder E. M., Ramel A. (2006). A population-based study of exclusive breastfeeding in Icelandic women: is there a relationship with depressive symptoms and parenting stress?. *International Journal of Nursing Studies*.

[80] Ystrom E. (2012). Breastfeeding cessation and symptoms of anxiety and depression: a longitudinal cohort study. *BMC Pregnancy and Childbirth*.

[81] Flores-Quijano M. E., Córdova A., Contreras-Ramírez V., Farias-Hernández L., Cruz Tolentino M., Casanueva E. (2008). Risk for postpartum depression, breastfeeding practices, and mammary gland permeability. *Journal of Human Lactation*.

[82] Clifford T. J., Campbell K. M., Speechley K. N., Gorodzinsky F. (2006). Factors influencing full breastfeeding in a Southwestern Ontario community: assessments at 1 week and at 6 months postpartum. *Journal of Human Lactation*.

[83] Kendall-Tackett K., Cong Z., Hale T. W. (2013). Depression, sleep quality, and maternal well-being in postpartum women with a history of sexual assault: a comparison of breastfeeding, mixed-feeding, and formula-feeding mothers. *Breastfeeding Medicine*.

[84] Hahn-Holbrook J., Haselton M. G., Dunkel Schetter C., Glynn L. M. (2013). Does breastfeeding offer protection against maternal depressive symptomatology?: a prospective study from pregnancy to 2 years after birth. *Archives of Women's Mental Health*.

[85] Bogen D. L., Hanusa B. H., Moses-Kolko E., Wisner K. L. (2010). Are maternal depression or symptom severity associated with breastfeeding intention or outcomes?. *Journal of Clinical Psychiatry*.

[86] Chung E. K., McCollum K. F., Elo I. T., Lee H. J., Culhane J. F. (2004). Maternal depressive symptoms and infant health practices among low-income women. *Pediatrics*.

[87] Cox J. L., Connor Y., Kendell R. E. (1982). Prospective study of the psychiatric disorders of childbirth. *British Journal of Psychiatry*.

[88] Josefsson A., Angelsiöö L., Berg G. (2002). Obstetric, somatic, and demographic risk factors for postpartum depressive symptoms. *Obstetrics and Gynecology*.

[89] Lau Y., Chan K. S. (2007). Influence of intimate partner violence during pregnancy and early postpartum depressive symptoms on breastfeeding among chinese women in Hong Kong. *Journal of Midwifery and Women's Health*.

[90] O'Neill T., Murphy P., Greene V. T. (1990). Postnatal depression—aetiological factors. *Irish Medical Journal*.

[91] Ramsay M., Gisel E. G., McCusker J., Bellavance F., Platt R. (2002). Infant sucking ability, non-organic failure to thrive, maternal characteristics, and feeding practices: a prospective cohort study. *Developmental Medicine & Child Neurology*.

[92] Dennis C.-L., McQueen K. (2009). The relationship between infant-feeding outcomes and postpartum depression: a qualitative systematic review. *Pediatrics*.

[93] Galler J. R., Harrison R. H., Ramsey F., Chawla S., Taylor J. (2006). Postpartum feeding attitudes, maternal depression, and breastfeeding in Barbados. *Infant Behavior and Development*.

[94] Tamminen T. (1988). The impact of mother's depression on her nursing experiences and attitudes during breastfeeding. *Acta Paediatrica Scandinavica. Supplement*.

[95] Dennis C. L. (2003). The breastfeeding self-efficacy scale: psychometric assessment of the short form. *Journal of Obstetric, Gynecologic, & Neonatal Nursing*.

[96] Dai X., Dennis C.-L. (2003). Translation and validation of the breastfeeding self-efficacy scale into Chinese. *Journal of Midwifery and Women's Health*.

[97] Field T., Diego M., Hernandez-Reif M., Figueiredo B., Ezell S., Siblalingappa V. (2010). Depressed mothers and infants are more relaxed during breastfeeding versus bottlefeeding interactions: brief report. *Infant Behavior and Development*.

[98] Edhborg M., Matthiesen A.-S., Lundh W., Widström A.-M. (2005). Some early indicators for depressive symptoms and bonding 2 months postpartum—a study of new mothers and fathers. *Archives of Women's Mental Health*.

[99] Matthiesen A.-S., Ransjö-Arvidson A.-B., Nissen E., Uvnäs-Moberg K. (2001). Postpartum maternal oxytocin release by newborns: effects of infant hand massage and sucking. *Birth*.

[100] Skalkidou A., Hellgren C., Comasco E., Sylvén S., Sundström Poromaa I. (2012). Biological aspects of postpartum depression. *Women's Health*.

[101] Viero C., Shibuya I., Kitamura N. (2010). Oxytocin: crossing the bridge between basic science and pharmacotherapy. *CNS Neuroscience & Therapeutics*.

[102] Figueiredo B., Dias C. C., Brandão S., Canário C., Nunes-Costa R. (2013). Breastfeeding and postpartum depression: state of the art review. *Jornal de Pediatria*.

[103] Tu M. T., Lupien S. J., Walker C.-D. (2006). Diurnal salivary cortisol levels in postpartum mothers as a function of infant feeding choice and parity. *Psychoneuroendocrinology*.

[104] Stuebe A. M., Grewen K., Pedersen C. A., Propper C., Meltzer-Brody S. (2012). Failed lactation and perinatal depression: common problems with shared neuroendocrine mechanisms?. *Journal of Women's Health*.

[105] Murray L., Carothers A. D. (1990). The validation of the Edinburgh Post-natal depression scale on a community sample. *The British Journal of Psychiatry*.

